# Supplementary material for: The Systems Biology Research Tool: evolvable open-source software
Source: BMC Syst Biol. 2008 Jun 29;2:55. doi: 10.1186/1752-0509-2-55 (PMC2446383; doi:10.1186/1752-0509-2-55)
Supplement: Additional file 1 — SBRT Archive. An archive of the current version of the Systems Biology Research Tool. [file 1752-0509-2-55-S1.zip › sbrt-1.4.0/doc/developers_guide/api/sbrt/shell/text/MapEntryFormat.html]

MapEntryFormat


|  |  |  |
| --- | --- | --- |
| **Overview** \| **Package** \| **Class** \| **Use** \| **Tree** \| **Deprecated** \| **Index** \| **Help** | |  |
| **PREV CLASS**   **NEXT CLASS** | **FRAMES**    **NO FRAMES**     **All Classes** |  |
| SUMMARY: NESTED \| FIELD \| CONSTR \| METHOD | DETAIL: FIELD \| CONSTR \| METHOD |  |


---


## sbrt.shell.text Interface MapEntryFormat<K,V>

**Type Parameters:**: `K` - the key type.: `V` - the value type.

**All Superinterfaces:**: Format

**All Known Implementing Classes:**: AbstractMapEntryFormat, ColonMapEntryFormatV1, EqualsMapEntryFormatV1

---

``` public interface MapEntryFormat<K,V> extends Format ```

This interface is used to represent formats for map entries.

**Author:**
:   This interface was written and documented by
    Jeremiah Wright while in the Wagner lab.

---

| **Method Summary** | |
| --- | --- |
| `<S extends K> java.lang.String` | `formatKey(S key)`             Returns a formatted string representation of the provided key. |
| `java.lang.String` | `formatPair(java.util.Map.Entry<? extends K,? extends V> keyValuePair)`             Returns a formatted string representation of the provided key-value pair. |
| `<S extends K,T extends V> java.lang.String` | `formatPair(S key, T value)`             Returns a formatted string representation of the provided key-value pair. |
| `<T extends V> java.lang.String` | `formatValue(T value)`             Returns a formatted string representation of the provided value. |
| `K` | `parseKey(java.lang.String key)`             Parses the provided string and returns its corresponding key. |
| `java.util.Map.Entry<K,V>` | `parsePair(java.lang.String keyValuePair)`             Parses the provided string and returns a corresponding map entry. |
| `V` | `parseValue(java.lang.String value)`             Parses the provided string and returns its corresponding value. |

**Method Detail**

### formatKey

```
<S extends K> java.lang.String formatKey(S key)
```

:   Returns a formatted string representation of the
    provided key.

    :   **Type Parameters:**: `S` - a type that extends the primary key type of this format. **Parameters:**: `key` - the key to be formatted. **Returns:**: a formatted string representation of the provided key.

---


### formatValue

```
<T extends V> java.lang.String formatValue(T value)
```

:   Returns a formatted string representation of the
    provided value.

    :   **Type Parameters:**: `T` - a type that extends the primary value type of this format. **Parameters:**: `value` - the value to be formatted. **Returns:**: a formatted string representation of the provided value.

---


### formatPair

```
<S extends K,T extends V> java.lang.String formatPair(S key,
                                                      T value)
```

:   Returns a formatted string representation of the
    provided key-value pair.

    :   **Type Parameters:**: `S` - a type extending the primary key type.: `T` - a type extending the primary value type. **Parameters:**: `key` - the key.: `value` - the value. **Returns:**: a formatted string representation of the provided key-value pair.

---


### formatPair

```
java.lang.String formatPair(java.util.Map.Entry<? extends K,? extends V> keyValuePair)
```

:   Returns a formatted string representation of the
    provided key-value pair.

    :   **Parameters:**: `keyValuePair` - the key-value pair to be formatted. **Returns:**: a formatted string representation of the provided key-value pair.

---


### parseKey

```
K parseKey(java.lang.String key)
```

:   Parses the provided string and returns its
    corresponding key.

    :   **Parameters:**: `key` - a string representation of a key. **Returns:**: the key corresponding to the provided string.

---


### parseValue

```
V parseValue(java.lang.String value)
```

:   Parses the provided string and returns its
    corresponding value.

    :   **Parameters:**: `value` - a string representation of a value. **Returns:**: the value corresponding to the provided string.

---


### parsePair

```
java.util.Map.Entry<K,V> parsePair(java.lang.String keyValuePair)
```

:   Parses the provided string and returns a
    corresponding map entry.

    :   **Parameters:**: `keyValuePair` - a string representation of a key-value pair, or in other words, a map entry. **Returns:**: a map entry containing the key-value pair corresponding to the provided string.


---


|  |  |  |
| --- | --- | --- |
| **Overview** \| **Package** \| **Class** \| **Use** \| **Tree** \| **Deprecated** \| **Index** \| **Help** | |  |
| **PREV CLASS**   **NEXT CLASS** | **FRAMES**    **NO FRAMES**     **All Classes** |  |
| SUMMARY: NESTED \| FIELD \| CONSTR \| METHOD | DETAIL: FIELD \| CONSTR \| METHOD |  |


---
